# A broadband integrated microwave photonic mixer based on balanced photodetection

**DOI:** 10.1007/s12200-023-00064-5

**Published:** 2023-05-26

**Authors:** Zhenzhu Xu, Li Mei, Yuhua Chong, Xudong Gao, Shoubao Han, Chengkun Yang, Lin Li

**Affiliations:** 1 Anhui Province Engineering Laboratory for Antennas and Microwave, East China Research Institute of Electronic Engineering, Hefei, 230000 China; 2 Representative Office of the Army Armament Department in Hefei District, Hefei, 230000 China

**Keywords:** Microwave photonics, Integrated photonics, Frequency conversion, Photonic mixer, Balanced detection

## Abstract

An integrated microwave photonic mixer based on silicon photonic platforms is proposed, which consist of a dual-drive Mach–Zehnder modulator and a balanced photodetector. The modulated optical signals from microwave photonic links can be directly demodulated and down-converted to intermediate frequency (IF) signals by the photonic mixer. The converted signal is obtained by conducting off-chip subtraction of the outputs from the balanced photodetector, and subsequent filtering of the high frequency items by an electrical low-pass filter. Benefiting from balanced detection, the conversion gain of the IF signal is improved by 6 dB, and radio frequency leakage and common-mode noise are suppressed significantly. System-level simulations show that the frequency mixing system has a spurious-free dynamic range of 89 dB·Hz^2/3^, even with deteriorated linearity caused by the two cascaded modulators. The spur suppression ratio of the photonic mixer remains higher than 40 dB when the IF varies from 0.5 to 4 GHz. The electrical-electrical 3 dB bandwidth of frequency conversion is 11 GHz. The integrated frequency mixing approach is quite simple, requiring no extra optical filters or electrical 90° hybrid coupler, which makes the system more stable and with broader bandwidth so that it can meet the potential demand in practical applications.

**Graphical Abstract:**

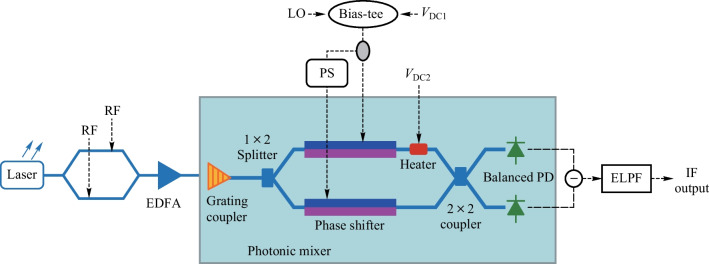

## Introduction

A microwave mixer is an indispensable module in a variety of electronic systems such as radar, satellite, radio communication, and electronic warfare systems, which realizes a down-conversion of radio frequency (RF) signal to an intermediate frequency (IF) to ensure further signal processing. Microwave mixing has also been widely applied in vector signal modulation and demodulation [1], frequency synthesis [2], and in frequency and phase discrimination [3, 4]. With the rapid development of electronic systems, the demand is increasing for systems with characteristics of high-frequency band, large bandwidth, large dynamic range and multifunction integration. However, electrical mixers are facing an electronic bottleneck due to factors such as poor RF/LO isolation, electromagnetic interference, and bandwidth constraint, hence they can hardly meet the demand of rapid development of electronic system [5]. Microwave photonic mixers outperform traditional electrical mixers in terms of instantaneous bandwidth, electromagnetic interference, insertion loss, and RF/LO isolation [6]. Moreover, the microwave photonics mixer is compatible with other microwave photonic signal generation, transmission and processing systems. Due to these advantages, photonic mixers have gained much attention and intensive research [7–14].

In the past decades, various approaches have been proposed to realize the microwave photonic mixer. For example, a filter-free photonic microwave single sideband mixer was demonstrated by using a dual-parallel Mach–Zehnder modulator (DPMZM) and a 90° hybrid coupler [10]. The RF and local oscillator (LO) signals pass through the same 90° hybrid coupler, hence the isolation between the RF and LO signals deteriorated significantly. To achieve high RF/LO isolation and spurs suppression, other approaches have been also presented to realize microwave photonic mixers, such as the methods based on a dual-polarization modulator [11], a DPMZM followed by optical bandpass filter (OBPF) [12, 13], as well as a dual-polarization DPMZM modulator [14]. However, these approaches are either based on a polarization modulator which is sensitive to environmental perturbations, or requiring an optical filter which suffers from the limited operation bandwidth owing to the small slope of the filter. Moreover, these above-mentioned frequency conversion architectures are all based on discrete components. There have been few reports of integrated microwave photonic mixer systems [15, 16].

Integrated photonic mixers provide promising solutions for many electronic systems such as avionic platforms which have strict requirements regarding weight and size. Additionally, the integration approaches can improve the system stability compared to those based on discrete components [17]. In this paper, an integrated photonic mixer based on silicon photonic (SiP) platforms is proposed. The integrated photonic mixer are mainly comprised of a dual-drive Mach–Zehnder modulators (MZM) and a balanced photodetector (BPD). Frequency mixing sysytem with high isolation between RF and LO signals is achieved based on cascaded MZMs. The analog metrics such as spurious-free dynamic range (SFDR), mixing spur suppression, frequency response of the integrated photonic mixer are carefully investigated. Benefiting from balanced detection, the IF gain and SFDR of the proposed frequency mixing system are improved, compared to the case with a regular photodetector (PD). This integrated frequency mixing approach is quite simple, optical filter-free and stable, which meets the application requirements of analog optical links.

## Configuration and principle

### Architecture of the proposed mixer

**Fig. 1:**
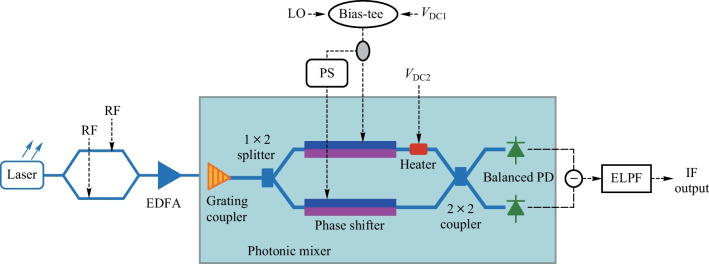
Schematic diagram of the RF mixing system based on the proposed integrated photonic mixer. *EDFA* erbium-doped fiber amplifier, *BPD* balanced photodetector, *ELPF* electrical low-pass filter

A schematic diagram of the RF mixing system based on the proposed integrated photonic mixer is shown in Fig. 1. The architecture of the integrated photonic mixer is marked in pale blue background. Thanks to the SiP integration platform, the proposed photonic mixer has a high level of integration. As shown in Fig. 1, the photonic mixer consists of a grating coupler, a SiP MZM, a 2 × 2 multimode interference (MMI) coupler, and a germanium-on-silicon BPD on-chip. The grating coupler is used to inject optical signal from an optical fiber into the system. The SiP MZM contains two PN junctions to implement electro-optical (EO) modulation, which can work in a single-drive mode or dual-drive mode. The heater placed after the PN junction in one arm of the MZM enables control of bias point of the MZM. Outputs of the MZM are then combined through a 2 × 2 MMI and input into the BPD. The BPD consists of two identical detectors, and subtraction between the two outputs of the BPD are conducted to obtain the frequency down-converted signal off-chip, while each output of the BPD can be separately tested for comparison.

The integrated photonic mixer was fabricated in CUMEC Photonics process. A coplanar waveguide traveling wave electrode with GSGSG (G = ground, S = signal) RF pad configuration was employed in the MZM. Resistors with impedance of 50 Ω terminated each traveling wave electrode. According to the CUMEC Photonics PDK, the *V*_π_·*L* of the MZM is 1.8 V·cm, and the 3 dB EO bandwidth is more than 20 GHz at − 2 V reverse bias. The PD exhibits a bandwidth of 20 GHz and responsivity of 0.9 A/W at − 1 V reverse bias.

### Principle of the photonic-based frequency mixing

In the photonic-based frequency mixing system as shown in Fig. 1, the optical carrier sent to the frequency mixing system is expressed as $E_{0} (t) = E_{0} {\text{e}}^{{{\text{j}}w_{0} t}}$, where *E*_0_ and *ω*_0_ are the amplitude and angular frequency of the optical carrier, respectively. Supposing that the applied RF signal feeding to the first MZM (MZM1) is $\upsilon_{{{\text{RF}}}} (t) = V_{{{\text{RF}}}} \sin (\omega_{{{\text{RF}}}} t)$, where *V*_RF_ and *ω*_RF_ are the amplitude and angular frequency of the RF signal, respectively. To maximize the modulation efficiency, the MZM1 is worked at the quadrature point, which implies that the static phase difference due to the different reverse bias of the PN junctions and the heater is π/2. To improve the linearity, the SiP MZM is operated under a differential drive [18–20]. After passing through the MZM1, the field of output optical signal can be written as

1 $$E_{{{\text{RF}}}} (t) = \tfrac{{E_{0} {\text{e}}^{{{\text{j}}w_{0} t}} }}{2}\left\{ {{\text{e}}^{{ - \alpha_{{\text{A}}} }} {\text{e}}^{{{\text{j}}[\beta \sin (\omega_{{{\text{RF}}}} t) + {\uppi }/2]}} + {\text{e}}^{{ - \alpha_{{\text{B}}} }} {\text{e}}^{{ - {\text{j}}\beta \sin (\omega_{{{\text{RF}}}} t)}} } \right\},$$

where $\alpha_{{\text{A}}}$ and $\alpha_{{\text{B}}}$ are attenuation coefficients of the upper and lower arms, respectively. $\beta = {{{\uppi }V_{{{\text{RF}}}} } \mathord{\left/ {\vphantom {{{\uppi }V_{{{\text{RF}}}} } {V_{{\uppi }} }}} \right. \kern-0pt} {V_{{\uppi }} }}$ is the modulation index of the RF signal, in which $V_{{\uppi }}$ is the half-wave voltage of the SiP MZM. Note that the nonlinear EO response and absorption loss in the two arms are neglected in Eq. (1). When absorption loss in the SiP MZM is neglected, Eq. (1) can be simplified as

2 $$E_{{{\text{RF}}}} (t) = \tfrac{{E_{0} {\text{e}}^{{{\text{j}}w_{0} t}} }}{2}\left\{ {{\text{e}}^{{{\text{j}}[\beta \sin (\omega_{{{\text{RF}}}} t) + {\uppi }/2]}} + {\text{e}}^{{ - {\text{j}}\beta \sin (\omega_{{{\text{RF}}}} t)}} } \right\}.$$

The modulated signal is then coupled into the integrated photonic mixer through a grating coupler and equally divided into the two arms of the SiP MZM (MZM2) via a 1 × 2 splitter. The MZM2 in photonic mixer is biased at the maximum transmission point and operated also under a differential drive. After being modulated by the LO signal, the optical signals in the two arms of the MZM2 combine through a 2 × 2 MMI and then received by the BPD. It is known that for a 2 × 2 MMI, when light field is input from one port, the phase of the two output signal should theoretically differ by π/2. Assuming that the LO signal feeding into the MZM2 on chip is $\upsilon_{{{\text{LO}}}} (t) = V_{{{\text{LO}}}} \sin (\omega_{{{\text{LO}}}} t)$, where the value of *V*_LO_ is equal to *V*_RF_. The optical field in the upper and lower output ports of the 2 × 2 MMI can be expressed as

3 $$\begin{gathered} E_{{{\text{out1}}}} (t) = \tfrac{{E_{0} {\text{e}}^{{{\text{j}}w_{0} t}} }}{4}\left\{ {{\text{e}}^{{{\text{j}}[\beta \sin (\omega_{{{\text{RF}}}} t) + {\uppi }/2]}} + {\text{e}}^{{ - {\text{j}}\beta \sin (\omega_{{{\text{RF}}}} t)}} } \right\} \cdot \left\{ {{\text{e}}^{{{\text{j}}\beta \sin (\omega_{{{\text{LO}}}} t)}} + {\text{e}}^{{ - {\text{j}}[\beta \sin (\omega_{{{\text{LO}}}} t) - {\uppi }/2]}} } \right\}, \hfill \\ E_{{{\text{out2}}}} (t) = \tfrac{{E_{0} {\text{e}}^{{{\text{j}}w_{0} t}} }}{4}\left\{ {{\text{e}}^{{{\text{j}}[\beta \sin (\omega_{{{\text{RF}}}} t) + {\uppi }/2]}} + {\text{e}}^{{ - {\text{j}}\beta \sin (\omega_{{{\text{RF}}}} t)}} } \right\} \cdot \left\{ {{\text{e}}^{{{\text{j}}[\beta \sin (\omega_{{{\text{LO}}}} t) + {\uppi }/2]}} + {\text{e}}^{{ - {\text{j}}\beta \sin (\omega_{{{\text{LO}}}} t)}} } \right\}, \hfill \\ \end{gathered}$$

where absorption loss and nonlinear EO response of the MZM2 in photonic mixer on chip are also neglected. The output signal after square-law detection by the two PDs in the BPD can be written as

4 $$\begin{aligned} I_{1} (t) &= \eta \tfrac{{P_{0} }}{4}\left\{ {1 - \sin [\beta \sin (\omega_{{{\text{RF}}}} t)]} \right\} \cdot \left\{ {1 + \sin [\beta \sin (\omega_{{{\text{LO}}}} t)]} \right\} \\ &= \eta \tfrac{{P_{0} }}{4}\left\{ {1 - \sin [\beta \sin (\omega_{{{\text{RF}}}} t)] + \sin [\beta \sin (\omega_{{{\text{LO}}}} t)] - \sin [\beta \sin (\omega_{{{\text{RF}}}} t)]\sin [\beta \sin (\omega_{{{\text{LO}}}} t)]} \right\} \\ &\propto \eta \tfrac{{P_{0} }}{2}\left\{ {1 - J_{1} (\beta )\sin (\omega_{{{\text{RF}}}} t) + J_{1} (\beta )\sin (\omega_{{{\text{LO}}}} t) - J_{1} (\beta )J_{1} (\beta )\cos [(\omega_{{{\text{RF}}}} - \omega_{{{\text{LO}}}} )t] + J_{1} (\beta )J_{1} (\beta )\cos [(\omega_{{{\text{RF}}}} + \omega_{{{\text{LO}}}} )t]} \right\}, \\ I_{2} (t) &= \eta \tfrac{{P_{0} }}{4}\left\{ {1 - \sin [\beta \sin (\omega_{{{\text{RF}}}} t)]} \right\} \cdot \left\{ {1 - \sin [\beta \sin (\omega_{{{\text{LO}}}} t)]} \right\} \\ &= \eta \tfrac{{P_{0} }}{4}\left\{ {1 - \sin [\beta \sin (\omega_{{{\text{RF}}}} t)] - \sin [\beta \sin (\omega_{{{\text{LO}}}} t)] + \sin [\beta \sin (\omega_{{{\text{RF}}}} t)]\sin [\beta \sin (\omega_{{{\text{LO}}}} t)]} \right\} \\ &\propto \eta \tfrac{{P_{0} }}{2}\left\{ {1 - J_{1} (\beta )\sin (\omega_{{{\text{RF}}}} t) - J_{1} (\beta )\sin (\omega_{{{\text{LO}}}} t) + J_{1} (\beta )J_{1} (\beta )\cos [(\omega_{{{\text{RF}}}} - \omega_{{{\text{LO}}}} )t] - J_{1} (\beta )J_{1} (\beta )\cos [(\omega_{{{\text{RF}}}} + \omega_{{{\text{LO}}}} )t]} \right\}, \\ \end{aligned}$$

where *η* denotes responsivity of the PD, and *J*1(*β*) is the Bessel function of the first kind. Subtraction of the two outputs is conducted off-chip, and the obtained photocurrent after balanced detection can be expressed as

5 $$\begin{aligned} I(t) &= I_{1} (t) - I_{2} (t) \\ &\propto \eta P_{0} \left\{ {J_{1} (\beta )\sin(\omega_{{{\text{LO}}}} t) - J_{1} (\beta )J_{1} (\beta )\cos[(\omega_{{{\text{RF}}}} - \omega_{{{\text{LO}}}} )t] + J_{1} (\beta )J_{1} (\beta )\cos[(\omega_{{{\text{RF}}}} + \omega_{{{\text{LO}}}} )t]} \right\}. \\ \end{aligned}$$

As can be seen from Eq. (5), the frequency components in the RF-converted signals are at $\omega_{{{\text{RF}}}} - \omega_{{{\text{LO}}}}$, $\omega_{{{\text{RF}}}} + \omega_{{{\text{LO}}}},$ $\omega_{{{\text{LO}}}}$, and other high frequencies, where the frequency component at $\omega_{{{\text{RF}}}} - \omega_{{{\text{LO}}}}$ is the down-converted IF signals. Fortunately, components at $\omega_{{{\text{RF}}}} + \omega_{{{\text{LO}}}}$, $\omega_{{{\text{LO}}}}$, and other extra high frequencies can be easily filtered out by an electrical low-pass filter (ELPF). Comparing Eq. (5) with Eq. (4), it can be found that the balanced detection can double the target IF photocurrent [21]. The IF conversion gain is defined as $G(\text{dB}) = 10\log_{10} \left[ {({{I_\text{IF} } \mathord{\left/ {\vphantom {{I_{IF} } {V_{{{\text{RF}}}} }}} \right. \kern-0pt} {V_{{{\text{RF}}}} }})^{2} R_{{{\text{in}}}} R_{{{\text{out}}}} } \right]$, where *I*_IF_, *R*_in_, and *R*_out_ denote IF photocurrent, input impedance and output impedance, respectively. Hence balanced detection can improve the IF conversion gain by 6 dB [22]. Moreover, Eq. (5) indicates that RF leakage and common-mode noise can be canceled out by balanced detection, and the signal-to-noise ratio of the system may be improved.

## Results and discussion

To test the basic functionality of the proposed photonic mixer on-chip, a system-level simulation of the configuration as shown in Fig. 1 was conducted, where measured parameters of the key components are used. In the simulation, the laser had an output power of 13 dBm and a wavelength of 1550 nm. The fiber-to-chip coupling loss was set to 3.5 dB. The frequency of the RF signal feeding to the MZM1 was 12 GHz. The modulated optical signal after the MZM1 was amplified by an erbium-doped fiber amplifier (EDFA) to compensate for fiber-chip coupling loss and MZM insertion loss. The LO signal with frequency of 10 GHz was applied to the MZM2 on the photonic mixer chip to generate a down-converted 2 GHz IF signal. The input powers of the RF and LO signal were 12.1 and 15.1 dBm, respectively. A Bias tee applied − 1 V reverse bias to the PN-junction in the two arms of the MZM2 on the photonic mixer and made them work in depletion state. The total power of the optical signals received by each PD was about 8.7 dBm and the responsivity of the PDs was set as 0.9 A/W.

**Fig. 2:**
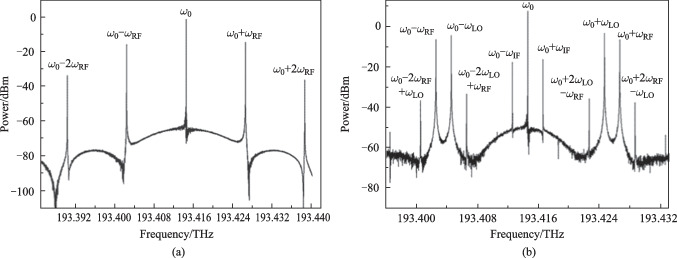
Output optical spectra of **a** the MZM1 and **b** the 2 × 2 MMI coupler

Figure 2 shows the output optical spectra after the MZM1 and 2 × 2 MMI. In the proposed photonic-based RF mixing system, the first off-chip MZM1 loads the RF signal with a differential drive mode and works at the quadrature point. As is shown in Fig. 2a, the output optical spectrum contains the optical carrier and positive and negative sidebands of the RF signal. Then the modulated optical signal couples into the MZM2 on-chip. The MZM2 on-chip is driven by the LO signal differentially and operates at the maximum transmission point. After being modulated by the LO signal, the optical signals in the two arms of the MZM2 in the photonic mixer combine through a 2 × 2 MMI. As the MZM in photonic mixer works at the maximum transmission point, the output optical spectrum of the 2 × 2 MMI coupler contains all positive and negative sidebands of the LO signal, as is shown in Fig. 2b.

**Fig. 3:**
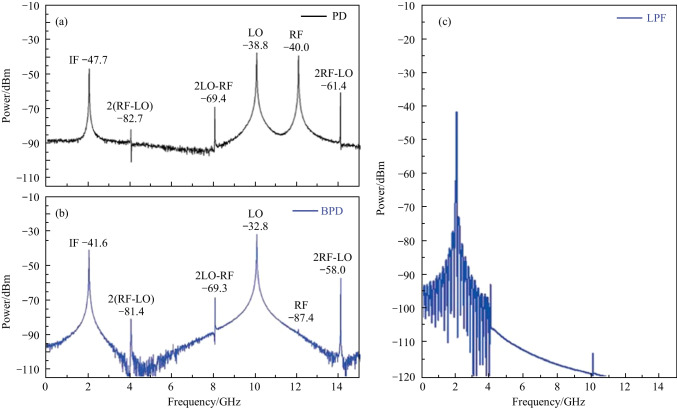
Simulated electrical spectra of the converted signals after **a** single PD, **b** BPD, and **c** 4 GHz ELPF

For balanced detection, subtraction between the two output electrical signals of BPD were conducted off-chip then measured by a spectrum analyzer. The electrical spectrum without balanced detection was obtained by connecting only one output port of the BPD to the spectrum analyzer. Figure 3a shows the results without balanced detection. As is shown, frequency components at *ω*_RF_ − *ω*_LO_ (2 GHz), *ω*_RF_ (12 GHz) and *ω*_LO_ (10 GHz) were generated. Due to the nonlinear effect of the SiP MZM, the second-order intermodulation distortion (IMD2, at 4 GHz) and third-order intermodulation distortions (IMD3, at 8 and 14 GHz) were also generated. For balanced detection, the simulated electrical spectrum is shown in Fig. 3b, where the desired IF component is improved by 6 dB and the RF leakage is significantly suppressed to − 87.4 dBm. Moreover, the output noise floor was evidently lowered after balanced detection. These results are consistent well with Eqs. (4) and (5). In this simulation, the power ratio of the desired IF and unwanted 2*ω*_IF_ (2IF) with the balanced detection is 40 dB. When the input power of the RF signal is decreased, the output power of 2IF component can be suppressed soon. The other mixing spurs and LO leakage in the high-frequency range can be easily excluded by an ELPF. Figure 3c shows the electrical spectra after an ELPF with cut-off frequency of 4 GHz. The low-pass filter strongly attenuates signals beyond 4 GHz.

**Fig. 4:**
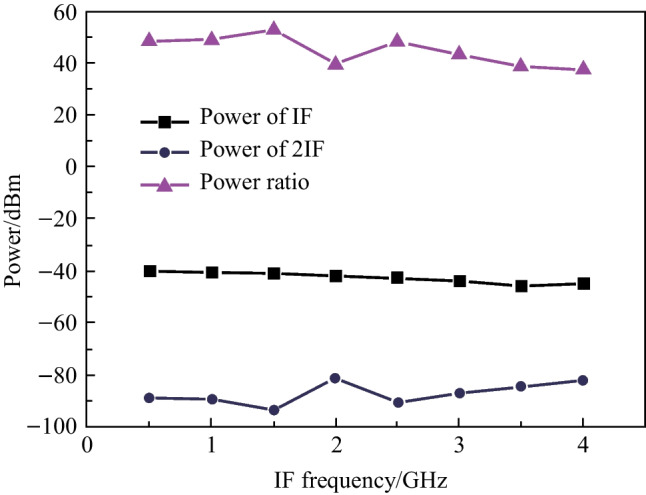
Mixing spur suppression results of the photonic mixer

It should be noted that the mixing spurs cannot be filtered by ELPF if the undesired interference component is close to the IF signal. Therefore, the 2IF suppression is important for achieving a high-performance frequency mixing system. To verify the mixing spurs suppression performance of this photonic mixer, the power ratio of the IF and 2IF signal in the case of balanced detection was measured. The RF frequency was tuned from 10.5 to 14.0 GHz in steps of 0.5 GHz, while the LO frequency was fixed at 10 GHz. The powers of RF and LO signal were set to 12.9 and 15.1 dBm, respectively. Figure 4 shows the mixing spur suppression results of the proposed frequency mixing system. It can be seen that the power ratios of the IF and 2IF signals remained approximately at or above 40 dB when the IF varies from 0.5 to 4 GHz.

**Fig. 5:**
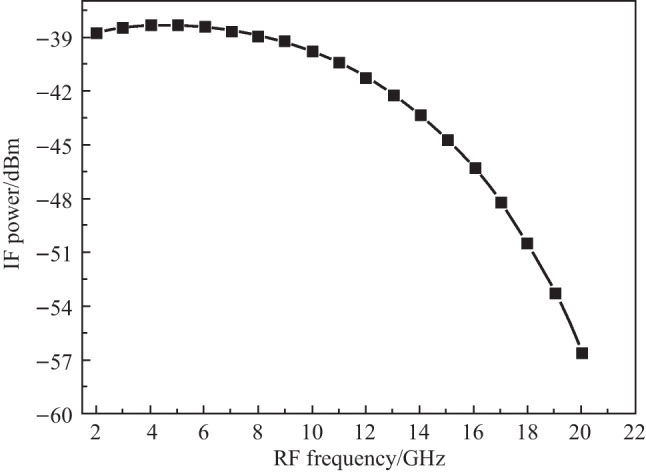
Simulated frequency responses of the photonic mixer

The frequency response of the frequency mixing system was tested in detail. The RF frequency was tuned from 10.5 to 14.0 GHz in steps of 1 GHz, and the IF frequency was fixed to 0.1 GHz by tuning the LO frequency accordingly. The obtained IF power as a function of input RF frequency is shown in Fig. 5. The output IF powers decrease significantly when the input RF frequency is higher than 12 GHz. The simulation predicts that the electrical-electrical 3 dB bandwidth of frequency conversion is 11 GHz, and the electrical-electrical 6 dB bandwidth is 15 GHz. In a photonic down-conversion system, the frequency conversion bandwidth is primarily determined by the bandwidth of the optical modulator since the down-conversion frequency received by PD is generally low.

**Fig. 6:**
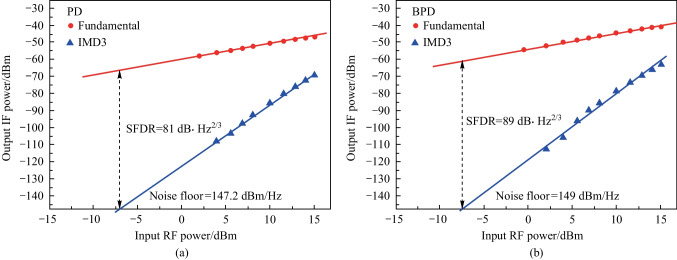
Power of fundamental signal (red dot) and IMD3 (blue triangle) as a function of input RF power **a** with out and **b** with balanced photodetection

Finally, the SFDR of the system with the configuration as shown in Fig. 1 was also investigated. SFDR is the power ratio of the fundamental signal to the IMD3 when the power of IMD3 is equal to the noise floor. In the simulation, nonlinear absorption behavior of the two cascaded SiP MZM was neglected. To model the nonlinearity of the phase shifters in this simulation, measured parameters of the SiP MZM was used. The laser was assumed to have a relative intensity noise of − 165 dB/Hz, and the PDs were assumed to have a thermal noise level of − 174 dBm/Hz. EDFA had a gain of 20 dB and noise figure of 4 dB. In this work, a two-tone RF signal at frequencies of 12.0 and 12.1 GHz were fed to the MZM1. Simultaneously, the LO signal with a frequency of 10 GHz was sent to the photonic mixer, and the power was set to 15.1 dBm. The optical power sent to the PD was about 8.7 dBm. Then the power of the fundamental signal (at 2 and 2.1 GHz) and the IMD3 signal (at 1.9 and 2.2 GHz) as a function of input RF power were measured. The results are shown in Fig. 6. As can be seen, the simulation predicts that the SFDR is 81 dB·Hz^2/3^ for the regular photodetection, and the noise floor is calculated to be 147.2 dBm/Hz. The IF output gain significantly increases by 6 dB for balanced photodetection, and the noise floor is reduced slightly to 149 dBm/Hz due to the suppressed common mode noise. Finally, balanced photodetection exhibits a higher SFDR of 89 dB·Hz^2/3^ compared to that of the regular photodetection.

In typical microwave photonic links, RF signals are modulated onto optical carrier by a modulator for transmitting in the optical fiber, and the modulated optical signals are then demodulated to RF signal by the PD after being transmitted through optical fiber [17]. The proposed on-chip photonic mixer is suitable for practical applications, as the received modulated optical signals from microwave photonic links can be directly demodulated and down-converted to IF signals [23, 24]. However, in most of the frequency conversion approaches based on dual-parallel modulator or dual-drive modulator, the modulated optical signal received from microwave photonic link must be demodulated to a RF signal before frequency down-conversion. Compared with these mixers, the proposed photonic mixer is quite simple, without any optical filters and electrical 90° hybrid coupler, which guarantees a broadband operation of the mixer. In addition, the integration capability of this structure will make the system more stable.

## Conclusions

An integrated photonic mixer is theoretically analyzed and numerically demonstrated. The architecture of integrated photonic mixeris comprised of a dual-drive MZM and a BPD on-chip. System-level simulations based on measured parameters of the key components are performed to demonstrate the performance of the photonic mixer. The analog metrics, such as the SFDR, mixing spur suppression, and frequency response of the integrated photonic mixer, are carefully investigated. The simulation results show that spur suppression ratios remain higher than 40 dB when the IF varies from 0.5 to 4 GHz. The electrical-electrical 3 dB bandwidth of frequency conversion is 11 GHz, and the electrical-electrical 6 dB bandwidth is 15 GHz. Benefiting from balanced detection, the IF gain is increased by 6 dB. The noise floor is lowered slightly due to the suppressed common mode noise. The photonic mixer exhibits a SFDR of 89 dB·Hz^2/3^. The proposed integrated photonic mixer with the advantage of simple structure and stable operation has great potential in various practical applications such as phased array radar and wireless communication.

## Data Availability

The data that support the findings of this study are available from the corresponding author, upon reasonable request.

## References

[1] Li J, Xiao J, Song X, Zeng Y, Yin C, Lv Q, Fan Y, Yin F, Dai Y, Xu K (2017). Full-band direct-conversion receiver with enhanced port isolation and I/Q phase balance using microwave photonic I/Q mixer. Chin. Opt. Lett..

[2] Zhang T, Wu K, Xiang J, Liu S, Chen J (2020). Highly reconfigurable microwave photonic waveform generation based on time-wavelength interleaving. IEEE Photonics J..

[3] Zhang K, Qin H, Zhao S, Li X, Lin T, Jiang W, Wang G (2019). Photonic approach to wideband Doppler frequency shift estimation system based on a DPMZM and a Sagnac Loop. Optik (Stuttg.).

[4] Burla M, Wang X, Li M, Chrostowski L, Azaña J (2016). Wideband dynamic microwave frequency identification system using a low-power ultracompact silicon photonic chip. Nat. Commun..

[5] Lin T, Zhang Z, Liu J, Zhao S, Li J, Zou C, Wang J, Zhang K, Jiang W (2020). Reconfigurable photonic microwave mixer with mixing spurs suppressed and dispersion immune for radio-over-fiber system. IEEE Trans. Microw. Theory Tech..

[6] Chen X, Li W, Yao J (2013). Microwave photonic link with improved dynamic range using a polarization modulator. IEEE Photonics Technol. Lett..

[7] Shi Z, Zhu S, Li M, Zhu N, Li W (2018). Reconfigurable microwave photonic mixer based on dual-polarization dual-parallel Mach–Zehnder modulator. Opt. Commun..

[8] Chen H, Chan E (2021). Microwave photonic I/Q mixer with phase shifting ability. IEEE Photonics J..

[9] Keshavarz H, Hosseini SE, Jamshidi K, Plettemeier D (2021). Silicon photonic-based integrated microwave photonic reconfigurable mixer, phase shifter, and frequency doubler. J. Lightwave Technol..

[10] Tang Z, Pan S (2016). A filter-free photonic microwave single sideband mixer. IEEE Microw. Wirel. Compon. Lett..

[11] Xuan L, Zhao S, Zhu Z, Qu K, Lin T, Hu D (2017). A reconfigurable photonic microwave mixer using a dual-polarization modulator. Opt. Quant. Electron..

[12] Gao Y, Wen A, Jiang W, Fan Y, Zhou D, He Y (2017). Wideband photonic microwave SSB up-converter and I/Q modulator. J. Lightwave Technol..

[13] Wang Y, Li J, Tao Z, Wang D, Xu J, Xin Z, Yang D, Lu Y (2017). All-optical microwave photonic down converter with tunable phase shift. IEEE Photonics J..

[14] Wang Y, Xu J, Wang D, Zhou T, Yang D, Zhong X, Yang F (2018). Microwave photonic mixer with large mixing spurs suppression and high RF/LO isolation. Optik (Stuttg.).

[15] Bottenfield CG, Ralph SE (2020). High-performance fully integrated silicon photonic microwave mixer subsystems. J. Lightwave Technol..

[16] Li J, Yang S, Chen H, Chen M (2021). Hybrid microwave photonic receiver based on integrated tunable bandpass filters. Opt. Express.

[17] Marpaung D, Yao J, Capmany J (2019). Integrated microwave photonics. Nat. Photonics.

[18] Streshinsky M, Ayazi A, Xuan Z, Lim AE, Lo GQ, Baehr-Jones T, Hochberg M (2013). Highly linear silicon traveling wave Mach–Zehnder carrier depletion modulator based on differential drive. Opt. Express.

[19] Zhou Y, Zhou L, Zhu H, Wong C, Wen Y, Liu L, Li X, Chen J (2016). Modeling and optimization of single-drive push-pull silicon Mach&-Zehnder modulator. Photon. Res..

[20] Khilo A, Sorace CM, Kärtner FX (2011). Broadband linearized silicon modulator. Opt. Express.

[21] Hung Y, Bortnik B, Fetterman H (2007). Dynamic-range enhancement and linearization in electrooptically modulated coherent optical links. J. Lightwave Technol..

[22] Gao Y, Wen A, Zhang W, Jiang W, Ge J, Fan Y (2017). Ultra-wideband photonic microwave I/Q mixer for zero-IF receiver. IEEE Trans. Microw. Theory Tech..

[23] Pagán VR, Haas BM, Murphy TE (2011). Linearized electrooptic microwave downconversion using phase modulation and optical filtering. Opt. Express.

[24] Chan EH, Minasian RA (2013). High conversion efficiency microwave photonic mixer based on stimulated Brillouin scattering carrier suppression technique. Opt. Lett..

